# The Impact of the mKidney mHealth System on Live Donor Follow-Up Compliance: Protocol for a Randomized Controlled Trial

**DOI:** 10.2196/11000

**Published:** 2019-01-15

**Authors:** Macey L Henderson, Alvin G Thomas, Ann K Eno, Madeleine M Waldram, Jaclyn Bannon, Allan B Massie, Michael A Levan, Dorry L Segev, Adam W Bingaman

**Affiliations:** 1 Department of Surgery Johns Hopkins University School of Medicine Baltimore, MD United States; 2 Department of Acute and Chronic Care Johns Hopkins School of Nursing Baltimore, MD United States; 3 Department of Epidemiology Johns Hopkins Bloomberg School of Public Health Baltimore, MD United States; 4 United Network for Organ Sharing Richmond, VA United States; 5 The Texas Transplant Institute Methodist Specialty and Transplant Hospital San Antonio, TX United States

**Keywords:** app, follow-up, health care, kidney transplantation, mHealth, mobile phone, randomized controlled trial, protocol

## Abstract

**Background:**

Every year, more than 5500 healthy people in the United States donate a kidney for the medical benefit of another person. The Organ Procurement and Transplantation Network (OPTN) requires transplant hospitals to monitor living kidney donors (LKDs) for 2 years postdonation. However, the majority (115/202, 57%) of transplant hospitals in the United States continue to fail to meet nationally mandated requirements for LKD follow-up. A novel method for collecting LKD follow-up is needed to ease both the transplant hospital-level and patient-level burden. We built mKidney—a mobile health (mHealth) system designed specifically to facilitate the collection and reporting of OPTN-required LKD follow-up data. The mKidney mobile app was developed on the basis of input elicited from LKDs, transplant providers, and thought leaders.

**Objective:**

The primary objective of this study is to evaluate the impact of the mKidney smartphone app on LKD follow-up rates.

**Methods:**

We will conduct a two-arm randomized controlled trial (RCT) with LKDs who undergo LKD transplantation at Methodist Specialty and Transplant Hospital in San Antonio, Texas. Eligible participants will be recruited in-person by a study team member at their 1-week postdonation clinical visit and randomly assigned to the intervention or control arm (1:1). Participants in the intervention arm will receive the mHealth intervention (mKidney), and participants in the control arm will receive the current standard of follow-up care. Our primary outcome will be policy-defined complete (all components addressed) and timely (60 days before or after the expected visit date) submission of LKD follow-up data at required 6-month, 1-year, and 2-year visits. Our secondary outcome will be hospital-level compliance with OPTN reporting requirements at each visit. Data analysis will follow the intention-to-treat principle. Additionally, we will collect quantitative and qualitative process data regarding the implementation of the mKidney system.

**Results:**

We began recruitment for this RCT in May 2018. We plan to enroll 400 LKDs over 2 years and follow participants for the 2-year mandated follow-up period.

**Conclusions:**

This pilot RCT will evaluate the impact of the mKidney system on rates of LKD and hospital compliance with OPTN-mandated LKD follow-up at a large LKD transplant hospital. It will provide valuable information on strategies for implementing such a system in a clinical setting and inform effect sizes for future RCT sample size calculations.

**International Registered Report Identifier (IRRID):**

DERR1-10.2196/11000

## Introduction

### Need for Living Donor Kidney Transplantation

Almost 100,000 patients with end-stage renal disease (ESRD) are currently on the waitlist for a deceased donor kidney transplant (DDKT) in the United States. An additional 30,000 are added to the waitlist each year. In 2017, only 14,038 received a DDKT. Most patients who are listed for a kidney transplant today (who do not have a living donor) will have to wait 3-7 years to get an organ offer. Live donor kidney transplantation (LDKT) offers patients with ESRD a timely and therapeutic modality that has superior outcomes to DDKT and dialysis [1]. LDKT has been recognized and promoted as the best treatment option for patients with kidney failure by the American Society of Transplantation Living Donor Community of Practice in a consensus statement [2].

### Sequelae of Living Kidney Donation

Living kidney donors (LKDs) experience 50% nephron loss (ie, one kidney) following donor nephrectomy, with the immediate consequence of 25%-40% loss of renal reserve as measured by the glomerular filtration rate (GFR). The health risks of this loss of GFR at nephrectomy appear to be minimal for most LKDs, with an estimated lifetime risk of ESRD of 90 per 10,000 LKDs [3-5]. However, further GFR loss might be consequential for some LKDs in the long term, especially in the event of *de novo* disease [5,6]. Diabetes mellitus (DM), hypertension (HTN), and glomerulonephritis account for over 60% of documented cases of ESRD in the LKD population [7].

Moreover, there are racial disparities in postdonation outcomes. In a national study linking Organ Procurement and Transplantation Network/United Network for Organ Sharing (OPTN/UNOS) registry data with administrative data of a private US health insurer, Lentine et al found that African American LKDs have a 1.5-fold higher risk of HTN, a 2.3-fold higher risk of DM, and a 2.3-fold higher risk of chronic kidney disease (CKD) compared with Caucasian LKDs at 7 years postdonation [8]. Using the same linkage, Lentine et al found that this disparity persisted across age, sex, and biological relationship to the recipient. The adjusted incidence of any renal diagnosis was higher among African American LKDs compared with Caucasian LKDs (14.9% vs 9.0%; adjusted hazard ratio [aHR] 1.72; *P*=.002), including CKD (12.6% vs 5.5%; aHR 2.32), proteinuria (5.7% vs 2.5%; aHR 2.27), and nephrotic syndrome (1.3% vs 0.1%; aHR 15.7) [9]. Within 15 years, African American LKDs have a higher absolute risk of ESRD (74.7 cases per 10,000 LKDs) than Caucasian (22.7 per 10,000) or Hispanic LKDs (32.6 per 10,000) [4]. While the overall risk of developing a renal disease is low [4], follow-up and self-care management are important.

### Importance of Living Kidney Donor Follow-Up to Reduce Progression to Late-Stage Renal Disease

Long before *de novo* diseases cause CKD and ESRD, they manifest as hyperglycemia, elevated blood pressure, proteinuria, and hematuria. Routine laboratory tests can screen for these subclinical entities. Appropriate LKD follow-up might present an opportunity for early detection and control of DM, HTN, glomerulonephritis, and CKD, thus slowing CKD or ESRD progression. Routine screening is especially important for young donors who face many decades with reduced renal reserve and, thus, a higher lifetime risk of ESRD.

### Current Landscape of Living Kidney Donor Follow-Up

OPTN/UNOS has collected postdonation follow-up data on LKDs since 1999. However, LKD follow-up has remained consistently poor. This prompted a national policy that began requiring centers to collect these data beginning in 2013 [10,11]. OPTN/UNOS now requires transplant hospitals to collect and submit clinical data (the presence of HTN, diabetes, dialysis, kidney-related complications, recent hospitalizations, medical insurance status, income, and vital status) for 80% and laboratory data (serum creatinine and urine protein) for 70% of LKDs for 2 years postdonation (Multimedia Appendix 1). Transplant hospitals are required to collect these data from each LKD within a 120-day period (60 days before or after the 6-month, 1-year, or 2-year postdonation date) for each follow-up visit [10]. Implementation of this requirement has shown limited improvement. In a national study, we found that only 43% (87/202) of transplant hospitals met OPTN/UNOS-mandated 6-month, 1-year, and 2-year thresholds for LKDs who donated in 2013 [12]. In the face of barriers, such as cost, LKD inconvenience, and the burden of data collection [13,14], transplant hospitals lack the tools to improve LKD engagement.

### Appropriate Living Kidney Donor Follow-Up Might Improve Our Ability to Understand Long-Term Sequelae of Donation

Given the limitations of the current system of LKD follow-up, alternative approaches are of utmost urgency to enable the medical community to uphold its obligation to care for living organ donors. To date, knowledge of postoperative health outcomes has largely been limited to perioperative mortality, long-term survival, and ESRD risk prediction, accounting for differences among racial and ethnic minorities [8,15-19]. Because of the lack of national follow-up and long-term data, inferences on long-term donor morbidity have been limited primarily to risks of cardiovascular disease, CKD, and ESRD [5,20,21]. In addition, only limited pilot data are available on the effect of donation on the pathophysiology of cardiovascular disease; hence, more research is needed to better define the effects of donation on cardiovascular disease surrogates and clinical events [22].

### Benefits of mHealth Technology to Patients and Providers

As mobile phone use has changed the way providers communicate with patients and each other, there is a need to develop the science of mobile health (mHealth) [23,24]. mHealth apps designed for smartphones are perceived to offer considerable potential as tools to engage patients in chronic disease management [25]. mHealth technologies have been implemented in several chronic disease settings with promising results. Users of an mHealth system to promote self-management among patients with type-2 diabetes (mDAWN) experienced improved disease biomarkers and decreased health distress after using the app for a 3-month period [26]. Delivery of an mHealth intervention for the prevention of weight gain (TXT2BFiT) resulted in modest but sustained weight loss after 9 months [27]. Furthermore, mHealth technologies have shown promise in facilitating behavioral interventions to reduce cardiovascular risk factors such as smoking, physical inactivity, and suboptimal nutrition [28].

### Benefits of Patient Engagement

Growing evidence suggests that health care is more efficient and effective when patients are actively engaged in their treatment [29]. Engaged patients collaborate with their providers, are better treated with respect and dignity, receive information related to their care, and are involved in decision making [30]. LKDs who are better engaged and informed may be able to keep better track of their postdonation health and may benefit from being able to visualize and summarize their health information, receive guidance on preventive care, and communicate with health care providers and the transplant system.

### Preliminary Data

In formative research conducted at the Johns Hopkins Hospital, 95 of 100 LKDs reported owning a smartphone [31], which is consistent with Pew Research Center findings that 92% of adults in the United States owned a mobile phone in 2015 [32]. Among participants, 80% (80/100) thought that mHealth technology would be useful in completing follow-up [31]. A pilot study of 69 LKDs found that engagement through short message service (SMS) text messages exceeded 80% at 2 years postdonation, compared with only 20% using traditional follow-up engagement strategies (ie, telephone; electronic medical record, EMR; or patient portal). Most LKDs (97%) selected electronic communication (email or SMS text message) as their preferred method of postdonation communication with the study team, with no significant differences by sex or race [33]. These findings demonstrate the feasibility of using electronic communications, like mHealth, to improve existing methods of postdonation communication with LKDs.

### Innovation

#### Design and Development of a New Technology

An effective method of follow-up communication with LKDs that does not place an undue burden on either patients or providers, allows for the monitoring and tracking of surgical recovery milestones, and can detect the development of *de novo* kidney disease to intervene when possible is needed. We designed an mHealth platform to capitalize on the available computing power and technologies that can transform the reach of medical care and research [34].

#### Novel Approach to Improve a Health System Failure

SMS text messages, emails, and mHealth are promising new approaches to rectify the striking gap in regular postdonation medical care for LKDs. mHealth interventions have been evaluated in clinical trials for self-management support, weight management, and prevention and management of cardiovascular disease and diabetes in other populations [26-28,35-38]. An mHealth system to engage LKDs in postdonation follow-up care might improve transplant hospitals’ ability to achieve compliance with OPTN/UNOS-mandated reporting requirements and provide a link to critical preventive care for LKDs.

#### Benefit and Innovation of mHealth for Living Kidney Donor Follow-Up

Despite the recent proliferation of mHealth technologies, few are currently used in research studies [39]. The National Institutes of Health (NIH) strategic plan supports contributing to the mHealth evidence base because everyone can use this technology [40]. With donors from the UNOS Living Donor Committee and among informal conversations with LKDs on Facebook, we identified the following 5 primary benefits to an mHealth system for LKD engagement and follow-up: (1) *portability*: mHealth goes beyond point-of-care clinical diagnostics, thus following the LKD past transplant hospital visits; (2) *scalability*: mHealth platforms have been shown to be economical to scale [41], and with no current mechanism for reimbursement for required follow-up, transplant hospitals absorb the cost; (3) *rich data input through continuous data sampling*: devices and wearables are meant to integrate with daily functions making data collection convenient, which could make LKD follow-up automatic and seamless; (4) *personalization capacity*, and (5) *real-time data and feedback with the ability for automated analyses*. An mHealth system could provide LKDs with an opportunity to medically engage with the hospital where they donated a kidney, ask for medical record review, and have a built-in system to alert primary care health care providers when laboratory tests or blood pressure measurements become worrisome. Novel applications of inexpensive and automated electronic communication technologies, such as mHealth, could enhance patient follow-up and be applied to other patient populations. In an environment of spiraling health care costs where paperwork is administratively expensive and burdensome, this technology could find broad application.

**Figure 1:**
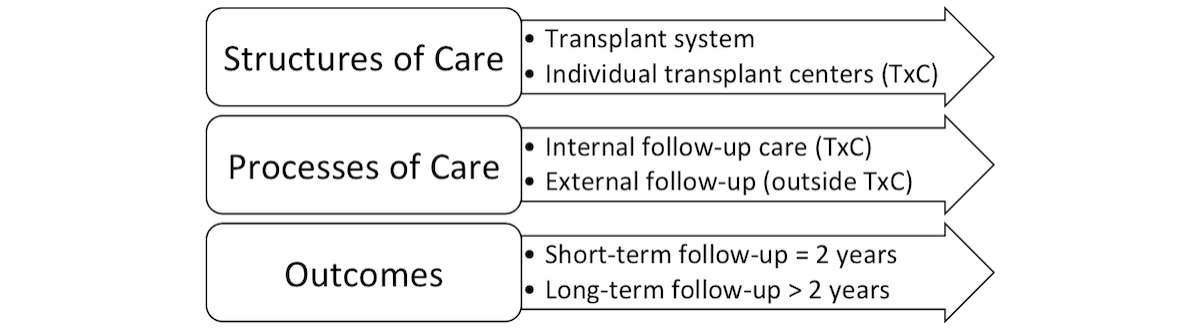
Adapted conceptual framework for living kidney donor follow-up.

### Overview and Theoretical Framework

This research is based on the Donabedian 3-factor conceptual framework of care quality, adapted to LKD follow-up (Figure 1) [42,43]. In the adapted conceptual framework, structures of care include the national organ transplant system and the transplant hospitals performing the living donor transplant surgery, which are responsible for reporting follow-up to OPTN/UNOS. Processes of care include LKD follow-up care that takes place both internal and external to the transplant hospital responsible for reporting. Outcomes include short-term (2-year) and long-term (>2-year) follow-up and vary in measures based on policy requirements and principles of prevention. The development of our mHealth system for LKD engagement and follow-up care sought to address all aspects of this framework.

### Objective

This study aims to pilot-test an mHealth system (mKidney) and design a future large-scale multicenter randomized controlled trial (RCT) of this intervention. We will recruit 400 participants (200 per year for 2 years) to pilot-test the intervention. We will compare rates of follow-up between LKDs in the intervention (mKidney system) and control arms (standard of care) at 6, 12, and 24 months to help estimate potential effect sizes of the intervention (to inform subsequent RCT design and power calculations).

## Methods

### Study Design

**Figure 2:**
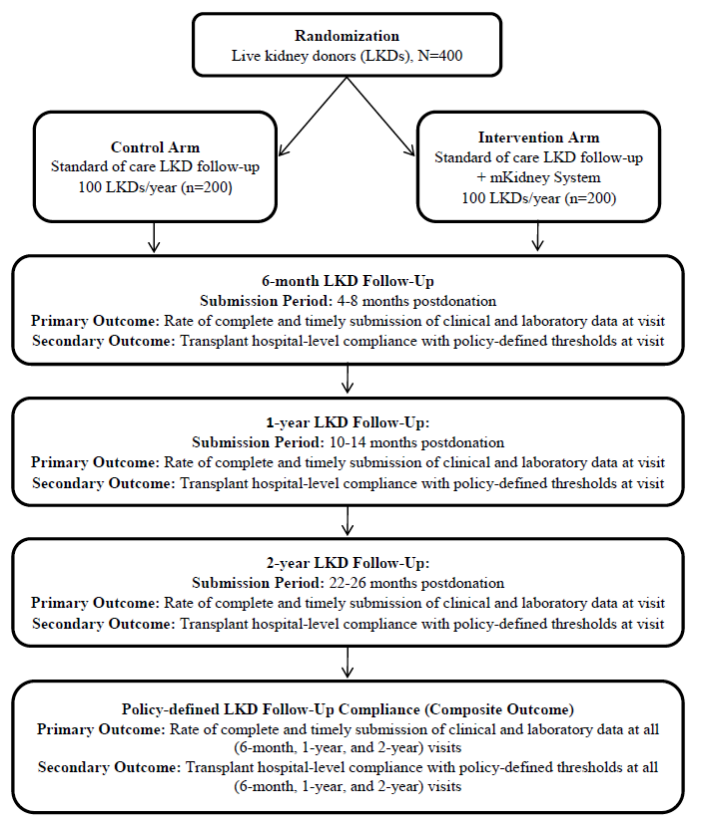
Schematic of the study design. LKD: living kidney donor.

We are conducting an exploratory pilot RCT with parallel-group design to evaluate the impact of the mKidney system on rates of postdonation follow-up among LKDs, in preparation for a fully powered clinical trial (NCT03400085). Participants will be randomized to the intervention (mKidney system) or control arm (standard of care) and will be followed for the mandated 2-year LKD follow-up period (Figure 2).

### Study Population

We plan to enroll 400 LKDs who have donated a kidney at Methodist Specialty and Transplant Hospital in San Antonio, Texas, during the study period. LKDs randomized to the intervention arm (approximate n=200) will receive the mKidney system, whereas LKDs randomized to the control arm (approximate n=200) will receive the current standard of follow-up care.

### Inclusion and Exclusion Criteria

For the pilot RCT, LKDs who have donated a kidney at the Methodist Specialty and Transplant Hospital during the study period will be eligible for study participation. We will exclude LKDs who do not speak English or own a smartphone device; by national policy, all donors are ≥18 years of age.

### mKidney System Description

The mKidney system includes 2 components—an LKD-facing smartphone app and a transplant provider-facing Web portal. Using the Health Insurance Portability and Accountability Act (HIPAA)-compliant mKidney app, LKDs can enter their responses to required questionnaires, record lab values, and submit a photo of their lab work at each 6-month, 1-year, and 2-year follow-up time point. The questionnaire for each follow-up time point will become available at the beginning of the 120-day submission period (Multimedia Appendix 1). LKDs will receive an automated SMS text message, email, and push notifications throughout the open submission period to prompt follow-up completion. If needed, transplant providers may contact LKDs using traditional engagement strategies (eg, telephone and EMR patient portal) in addition to automated mKidney app notifications. In addition, LKDs can access Web resources through the mKidney app, including the transplant hospital website and locations of laboratory testing sites. Using the secure, HIPAA-compliant mKidney Web portal, transplant providers can monitor patients’ compliance with follow-up, log additional contact attempts, view questionnaire responses, and export data for reporting purposes.

### Study Procedure

LKDs will undergo consent and randomization at their medically required 1-week postdonation clinical visit. Study personnel who have undergone Human Subjects Training will use a written consent form to document consent (Multimedia Appendix 2). Surgeon and clinician members of the study team will not participate in recruitment activities to avoid the potential for coercion and appearance of a conflict of interest. Paradata will be collected on the number of acceptances, eligible enrollments, and refusals.

We will assign participants either to the intervention (mKidney system) or the control arm (standard of care) using block randomization with block sizes ranging from 2 to 8. Block randomization will improve the probability of balanced groups over the course of the study. A statistician on the Johns Hopkins study team, blind to group allocations, will use this method to generate a list of sequential group assignments using Stata 15 for Linux (StataCorp Inc). The list will be used to create sequentially numbered, sealed envelopes that will be used to allocate consenting participants to the control or intervention arms of our study. Each patient will have a 50% chance of being assigned to the intervention arm of the study. Patients, health care workers on the study team, and study team members responsible for data collection and analysis will be aware of which arm participants are randomized to. Therefore, this study will not be blinded to providers, patients, or study personnel.

Study personnel will assist participants assigned to the mHealth intervention arm with downloading the mKidney app and explain its functioning. After enrollment in the study, participants in the intervention arm will receive notifications and have the ability to complete a questionnaire documenting their remote standard-of-care visit and enter the required laboratory values at 6 months, 1 year, and 2 years postdonation using the mKidney app. Participants in the control arm will be instructed to complete the required follow-up as is standard of care but will not use the mKidney app to do so (Figure 2).

The primary outcome of interest will be the rate of policy-defined complete (all components addressed) and timely (60 days before or after the expected visit date) submission of data at all 6-month, 1-year, and 2-year follow-up visits, compared between study arms. The secondary outcome will be the transplant hospital-level compliance with OPTN reporting requirements at each visit. Outcomes will be assessed independently for each follow-up time point and as a composite outcome over the study period, as well as will be compared between study arms following the intention-to-treat principle. To understand logistical or demographic barriers to implementation, we will also collect process data and utilize routinely collected data on LKDs in the study. These data include age, sex, race, ethnicity, and educational level of LKDs.

There will not be study-specific efforts to retain participants or promote the use of the mKidney app for LKD follow-up data submission, as this would be a form of intervention that might impact outcomes. However, transplant providers at Methodist Specialty and Transplant Hospital may contact LKDs for obtaining complete and timely LKD follow-up data to comply with nationally mandated follow-up requirements. Participants may withdraw from the RCT at any time without penalty. Withdrawal from the RCT would not preclude participants from obtaining regular medical care or follow-up care related to their kidney donation. If participants choose to withdraw, the study team will use the data collected prior to withdrawal and mark the remaining data as censored. Other than interventions that might impact the rates of LKD follow-up compliance, no concomitant care or interventions will be prohibited during the trial.

### Sample Size and Power Calculation

**Figure 3:**
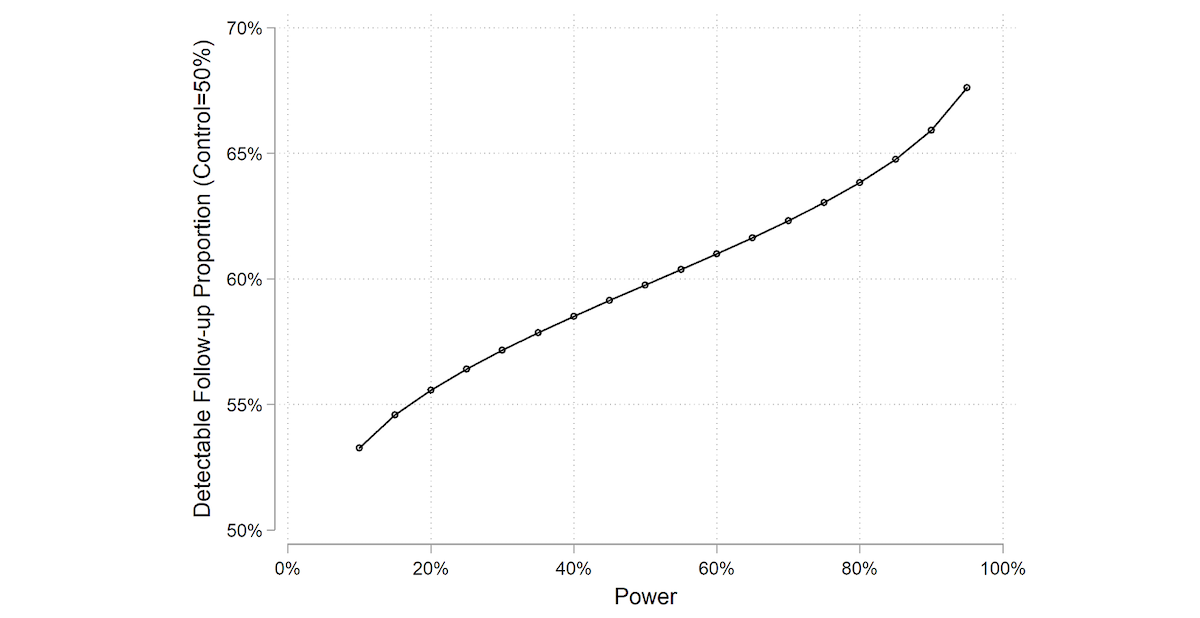
Power calculation of piloting mKidney with 200 donors per year.

If we recruit a total of 400 LKDs over a 2-year period and the proportion of control arm LKDs with compliant follow-up is 50%, we will have 80% power to detect a difference of 13.8% and 90% power to detect a difference of 15.9% (Table 1). If the projected follow-up rate of donors in the intervention arm is 67% (the minimum threshold for policy compliance), this study will have 79% power to detect a difference. If the follow-up rate in the intervention arm is 70%, this study will have 95% power to detect a difference (Figure 3). There is a possibility that we might face low levels of recruitment or high levels of dropout. If we are only able to recruit 300 LKDs over 2 years, then we will have 80% power to detect a difference of 15.9% and 90% power to detect a difference of 18.3%. If we are only able to recruit 200 LKDs over 2 years, we will have 80% power to detect a difference of 19.3% and 90% power to detect a difference of 22.1%.

**Table 1:** Power size calculations.

Number of recruited live kidney donors	Control proportion	Intervention proportion (80% power)	Delta (80% power)	Intervention proportion (90% power)	Delta (90% power)
200	0.50	0.693	0.193	0.721	0.221
300	0.50	0.659	0.159	0.683	0.183
400	0.50	0.638	0.138	0.659	0.159

### Analysis

Research team members at Johns Hopkins will conduct all analyses for this pilot RCT. Descriptive statistical methods will be used to analyze the frequency of key variables, including chi-square and rank-sum tests. Rates of the follow-up compliance among participants in the intervention and control arms will be compared using generalized linear regression. We will perform subgroup analyses for younger donors (age at donation<40 years), older donors (age ≥40 years), men, and women. In addition, the impact of the mKidney system on LKD follow-up compliance will be compared with historical follow-up with a difference-in-difference framework. All analyses will follow an intention-to-treat principle. Data will be analyzed with Stata 15 for Linux (StataCorp Inc).

### Ethics and Protection of Human Subjects

#### Ethical Standard

Participants are followed up for their compliance with standard-of-care recommendations, including a clinical visit (evaluating the vital status, income, medical insurance, recent hospitalizations, kidney-related complications, dialysis, HTN, and diabetes) and laboratory measurements (serum creatinine and urine protein levels). No additional care or procedures will be administered to study participants.

#### Institutional Review Board Approval

This study was reviewed and approved by both the Johns Hopkins School of Medicine Institutional Review Board (IRB) (IRB00162212) and Methodist Specialty and Transplant Hospital IRB (IRB12091661). Protocol amendments will be submitted to the Johns Hopkins School of Medicine and Methodist Specialty and Transplant Hospital IRBs.

#### Participant and Data Confidentiality

Only requisite study personnel at Methodist Specialty and Transplant Hospital will have access to identifying patient information in the EMR for extraction purposes. Johns Hopkins study team members will only be involved with data analysis and will have no direct patient contact in this study. Research team members at Johns Hopkins will receive data about whether patients enrolled in the pilot RCT at Methodist Specialty and Transplant Hospital completed their required 6-month, 1-year, and 2-year follow-up visits. All study personnel have received requisite training in data confidentiality and human subjects research.

LKD follow-up data will be stored on the emocha Mobile Health server for a minimum of 7 years according to HIPAA requirements. Research team members at Johns Hopkins will have access to the raw data submitted using the mKidney app and the system’s audit logs. All emocha platforms comply with HIPAA regulations on handling protected health information, including secure encryption of data, access controls, and industry-standard best practices. A robust role-based permission system limits system access to only authorized, authenticated users to ensure the need-to-know basis of protected health information.

#### Data Safety and Trial Monitoring

The Johns Hopkins School of Medicine IRB determined that a data monitoring committee was not necessary for this RCT due to minimal participant risk. Data monitoring will be conducted and reported by the Principal Investigator (PI) as projected by the data safety monitoring plan. The PI will immediately report any unanticipated adverse events or study deviations to the Johns Hopkins School of Medicine IRB. Trial conduct will be monitored through the mKidney system. Any viewing or modification of the system, or patient data, is logged in a persistent and unmodified database. Audit trail records include, but are not limited to, the action being taken, the date and time, and, in the case of modifications, both the old and new values. In addition, no data are ever deleted in the system; data are “soft-deleted” by marking with a flag that will hide the record during normal operations, but leaves it easily recoverable if needed.

## Results

We began recruitment for this pilot RCT in May 2018 at Methodist Specialty and Transplant Hospital in San Antonio, Texas. We plan to recruit for 2 years and to follow up participants for the 2-year mandated follow-up period. Pilot findings will inform the development of a larger, multisite proposal and will provide process measures, an initial comparison to standard or care, and will inform effect size estimation for a fully powered RCT.

## Discussion

### Potential Limitations and Proposed Solutions

#### Insufficient Recruitment

A potential challenge may be participant recruitment. While we anticipate high levels of participation, even with low recruitment, we believe the study will be feasible, given an expected living donor volume of approximately 800-1000 LKD transplants during the study period. The recruitment period can be extended if needed. If the living donor volume at the pilot transplant hospital is insufficient, we will leverage the existing study population, experienced research team, and resources associated with an ongoing NIH-funded cohort study of LKDs. These resources will help to ensure timeliness, feasibility, and a high likelihood of success. It is also possible that the effect size will be larger than the estimate in the power calculations and that a smaller sample might provide adequate power.

#### Special Populations

LKDs at Methodist Specialty and Transplant Hospital have historically been predominantly Caucasian and Hispanic; thus, recruitment of African Americans and Asian LKDs might be limited. We will consider age-related issues to technology adaptation and use, which could be a limitation to the implementation of the mKidney system. Based on recent trends at our pilot site, we anticipate approaching patients with a wide distribution of age.

#### Technical Infrastructure and Connectivity

We will leverage the robust resources of emocha Mobile Health Inc. and the Johns Hopkins University to limit possible challenges to the interoperability and functionality. Future updates to mobile operating systems or related software might affect the function of mKidney. We will continuously monitor the function of mKidney and provide updates as necessary with the developer, emocha Mobile Health.

#### Need for Tailoring for Differences in Adoption Among Different Racial and Ethnic Groups

Should we receive feedback that differs based on factors, such as sex, age, race or ethnicity, and health literacy, it might be necessary to tailor the mKidney system or design a different mHealth system to mitigate the potential for health disparities. The UNOS Living Donor Committee has expertise in the design, development, and cultural tailoring of transplant education materials and tools should the need arise to develop different versions.

### Dissemination Policy

Summary results of this pilot RCT will be reported to ClinicalTrials.gov no later than 1 year after the study completion date, as per the NIH Policy on Dissemination of NIH-Funded Clinical Trial Information [44]. We also anticipate submitting the findings of this pilot RCT for peer-reviewed publication. Authorship eligibility will be determined using the International Committee of Medical Journal Editors guidelines [45].

## Appendix

Multimedia Appendix 1 OPTN/UNOS Living Donor Follow-Up Worksheet.

Multimedia Appendix 2 Informed Consent Form.

## Abbreviations

CKD: chronic kidney disease
DDKT: deceased donor kidney transplant
DM: diabetes mellitus
EMR: electronic medical record
ESRD: end-stage renal disease
GFR: glomerular filtration rate
HTN: hypertension
IRB: Institutional Review Board
LKD: living kidney donor
mHealth: mobile health
NIH: National Institutes of Health
OPTN/UNOS: Organ Procurement and Transplantation Network/United Network for Organ Sharing
PI: Principal Investigator
RCT: randomized controlled trial
SMS: short service message
