# Supplementary material for: The Impact of the mKidney mHealth System on Live Donor Follow-Up Compliance: Protocol for a Randomized Controlled Trial
Source: JMIR Res Protoc. 2019 Jan 15;8(1):e11000. doi: 10.2196/11000 (PMC6350092; doi:10.2196/11000)
Supplement: Multimedia Appendix 2 [file resprot_v8i1e11000_app2.pdf]

CONSENT FOR RESEARCH
METHODIST HEALTHCARE SYSTEM

**Study Title:**	Pilot Study of a Mobile Health Intervention for Living Donor Follow-up
**Study #:**	IRB00162212
**Sponsor:**	Johns Hopkins University School of Medicine
**Study Doctor:**	Adam Bingaman, MD
**Telephone Number:**	(210) 575-8500
**NCT Number:**	**NCT03400085**

This form is written for your information.

INTRODUCTION

We are asking you to take part in a study using a mobile application. The purpose of this form is to give you information about:

- this study and
- the data collected

The form explains the:

- purpose
- procedures
- benefits and risks of the study

If you decide to take part in the study, we will ask you to sign this form. Taking part in this study is your choice. You do not have to take part if you do not want to. You can change your mind or stop your part in this study at any time.

Please read this form carefully. Ask as many questions as you need. This form may contain words you do not understand. The study staff will review this form with you. He or she will explain any words or procedures that you do not clearly understand. You should not sign this form if you have any questions that have not been answered to your satisfaction.

You may take home a copy of this form before you sign it. You should think about it before making your decision. You may discuss this study with family or friends before deciding. If you agree to take part in the study, please sign this form. We will give you a copy of this form. We will place a copy of this form in your medical records.

WHAT IS THE PURPOSE OF THE STUDY?

We are asking because you are going to have or have had a living related donor (LRD) kidney transplant. We are required to collect certain information about you and report it to the United Network for Organ Sharing (UNOS) for 2 years after your transplant. This collected information helps us provide better care for patients.

APPROVED
MARCH 09, 2018
Methodist Healthcare System
Institutional Review Board

This health information can be difficult to collect for transplant centers and new ways to collect this information are being tested. The mKidney application is one of the ways that we can collect this information by using smartphone technology.

This study is an information-gathering study only. Your doctor will manage your care no differently than he would if you were not part of this program. You do not need to take part in the study to receive treatment for your transplant.

If you agree to take part in the study, information about your disease and the treatment of your disease will be electronically collected for at least 2 years.

Once you sign this form, we will talk with you about your health history. We will review your past medical records. We will collect your medical data when you see your doctor at routine visits.

This data will be stored in an electronic database that is secure.

The type of data that will be collected during the study may include:

- Demographics - your age, gender, race, ethnicity
- Reason for kidney transplant, treatment regimen and dosing
- Medications currently taken and changes in medications
- Medical history
- Results of routinely conducted laboratory testing
- Hospitalizations
- Death

There will not be any extra visits to your doctor because of being in the study.

HOW MANY PEOPLE WILL TAKE PART IN THE STUDY?

All patients having a living related kidney donor transplant will be asked if they want to take part in the study. Up to 200 patients can take part in the study.

WHAT WILL HAPPEN DURING THE STUDY?

Patients who are getting a LRD kidney transplant will be invited to join the study. Patients who agree to be in the study will be randomized to use the mKidney application or to provide health information to the transplant center by the system currently in use. Randomization means, like the flip of a coin. You will get a 50/50 chance of being assigned to the mKidney application.

The study staff will assist you in downloading the application and instruct you how to enter your information and use the application. Patients that are not receiving the mKidney mobile application will be followed as standard of care.

APPROVED
MARCH 09, 2018
Methodist Healthcare System
Institutional Review Board

If you are using the mKidney application, you will be asked to enter your health information into the application after your 6-month follow up visit, 1-year follow up visit and 2-year follow up visit. We will ask you to enter the information within 60 days of your visit.

This information includes:

- Health questionnaire
- Laboratory values

ARE THERE ANY RISKS, SIDE EFFECTS AND DISCOMFORTS?

Medical Risks and Side Effects: There are no expected health risks related to taking part in the study. The study is about gathering information from your mKidney application. You will get the same care, even if you do not take part in the study.

POSSIBLE RISKS OF RELEASE OF PROTECTED HEALTH INFORMATION (PHI): The most important risk to you is that your PHI may not be kept totally private. PHI is any information about you and your health. This includes:

- health history, and
- information collected throughout the study and reported to UNOS

The possible loss of the privacy of your PHI could happen. However, this is rare. The study will follow all privacy and confidentiality laws that apply.

ARE THERE ANY BENEFITS?

You will not be helped by taking part in this study. What we learn may help doctors learn more about ways to improve LRD transplant long-term care.   This could help LRD transplant patients in the future.

WHAT ARE MY OTHER OPTIONS?

This study does not involve any treatment for your disease. You can choose not to be in the study.

WHAT ARE MY RIGHTS?

- Your part in the study is your choice.
- You do not have to take part in the study. We will still attempt to collect the required information for UNOS reporting.
- You can also withdraw at any time. There will be no penalty or loss of benefits. There also will be no change to your medical care.
- If you decide to leave the study, the sponsor will still use any information about you that has already collected. If you choose to withdraw from the study, please notify your doctor.

APPROVED
MARCH 09, 2018
Methodist Healthcare System
Institutional Review Board

We will tell you about any new findings. New findings may affect your decision to stay in the study.

Your doctor may end your part in the study if he/she thinks it is in your best interest. Your doctor or the sponsor may stop your part in the study at any time without your consent. This may occur if your doctor or the sponsor decides to end the study.

WHAT DO I HAVE TO DO TO TAKE PART IN THIS STUDY?

Your responsibilities as a study patient include the following:

- If you are assigned to the mKidney application, download the application to your electronic devices
- Enter remotely standard of care clinic visit questionnaire and laboratory values at 6 months, 1 year and 2 years after your transplant
- Coming to your regularly scheduled doctor's visits

ARE THERE ANY COSTS?

You will not have any extra costs from taking part in this study. The sponsor is paying for this study.

The Methodist Healthcare System will not provide funds for costs incurred because of taking part in this study.

WILL I BE PAID?

You will not be paid for being in this study.

Any product (like a new device application) or idea (including any intellectual property, such as patents) resulting from the study does not and will not belong to you. There is no plan for you to receive any financial compensation from the creation, use or sale of the data or any product or idea.

WHAT IF I AM INJURED DURING THIS STUDY OR HAVE AN EMERGENCY?

There are no additional benefits or free medical care provided because of the study. If you become injured or hurt during the course of the study, the sponsor does not have any plans to pay you or help pay for medical care if you are injured. You should not rely on any extra benefits or medical care from the study. The study is not intended to change your medical care.

Be aware that your health care payer/insurer might not cover the costs of study-related injuries or illnesses.

No funds have been set aside by the Methodist Healthcare System or by the study sponsor to provide to you in the event of injury. However, by signing this form, you are not giving up any legal rights to seek to obtain compensation for injury.

APPROVED
MARCH 09, 2018
Methodist Healthcare System
Institutional Review Board

WHO DO I CALL IF I HAVE QUESTIONS?

You may ask any questions about this study at any time. If you have more questions later or wish to report a non-emergency injury, please call:

Doctor	24 Hour Telephone
Adam Bingaman, MD	(210) 575-8500<br>1-800-298-7824
Methodist Clinical Trials Office	(210) 575-3161

The Methodist Healthcare System, Institutional Review Board (MHS IRB) has reviewed this study. MHS IRB is a group of people who review research studies to protect the rights and welfare of research participants. Review by MHS IRB does not mean that the study is without risks. You may contact the MHS IRB if you:

- have questions about your rights as a research participant
- are not able to resolve your concerns with your doctor
- have a complaint or
- have general questions about what it means to be in a research study

You may call the MHS IRB at (210) 575-4918 during normal business hours.

A description of this clinical trial will be available on http://www.clinicaltrials.gov as required by U.S. Law. This Web site will not include information that can identify you. At most, the Web site will include a summary of the results. You can search this Web site at any time.

AUTHORIZATION FOR USE AND DISCLOSURE OF INFORMATION

The purpose of this section is to make sure that you are properly informed of how your Protected Health Information (PHI) will be used or disclosed. Please read the information below carefully before signing this form.

You have the right to decide who can look at your Protected Health Information (PHI). Your PHI is any information that can reveal who you are. Examples of your PHI are:

- Name
- Age
- Date of birth

We will collect information about you:

- From your medical history and treatment before your transplant
- By asking you personally
- From your doctors
- From health care offices and hospitals

APPROVED
MARCH 09, 2018
Methodist Healthcare System
Institutional Review Board

Law protects your confidentiality. We will not use your name if this study is published in a medical journal or meeting. The sponsor or their representatives may inspect your study records and share as needed for the study. Study records that may identify you may include:

- original medical records
- signed consent form and
- other information

WHAT INFORMATION WILL BE USED OR DISCLOSED?

We may use and disclose your information related to your transplant as part of the study. Your PHI will be entered into the application and then the UNOS computer database. If you are assigned not to receive the mKidney application, you will be contacted by the transplant center and the data will be entered into the UNOS computer database per our regulatory requirements. It will be kept forever. Researchers and health care professionals will study the information in the database. The Sponsor plans to publish and share the results of the study.

WHO MAY USE AND DISCLOSE THE INFORMATION?

If you sign this form, the following may use or disclose the information described above:

- The study staff at John's Hopkins University School of Medicine
- The site's research staff and medical staff
- Any health care provider who provides services to you in connection with UNOS
- Any laboratories and other individuals and organizations that analyze your health information in connection with UNOS
- Methodist Healthcare System Institutional Review Board
- Local and federal regulatory agencies required by law to review the quality and safety of research including:
    - The U.S. Food and Drug Administration (FDA)
    - Department of Health and Human Services
    - Office for Human Research Protections
    - United Network for Organ Sharing
    - Other government agencies in the United States and other countries

SPECIFIC RIGHTS AND AUTHORIZATIONS

By signing this form, you authorize the use and/or disclosure of your PHI. The data given to the study may be given to the parties identified above. There is no plan for you to receive any payment from sharing your information.

The purpose for the uses and disclosures you are authorizing is to:

- conduct the research project described on this form and as explained to you
- ensure that the information relating to that research is available to all parties who may need it for research purposes

The results of the study may be published in a peer-reviewed journal.

APPROVED
MARCH 09, 2018
Methodist Healthcare System
Institutional Review Board

The study team may further give your PHI to groups not named here for study reasons. Once your PHI has been given to a third, party, federal privacy laws may no longer protect it. You have the right to see your study PHI. You can see your information at the end of this study. At that time, you can look at, copy and ask for corrections to your PHI. You can have this access for as long as the study staff keeps your study records.

You have a right to refuse to sign this form. If you do not sign the form, the following will not be affected:

- your health care outside the study
- payment for your health care and
- your health care benefits

You will not be able to take part in the study if you do not sign this form.

The authorization for use and disclosure of your information expires in 50 years. If you sign this form, you will have the right to cancel it at any time. You may withdraw your permission for the study team to collect, use and share your PHI at any time. You must send your request in writing to your study doctor at:

Methodist Healthcare System
Clinical Trials Office
7711 Louis Pasteur, Suite 708
San Antonio, TX 78229

If you withdraw your permission, your part in this study will end. We will stop collecting your medical information after we get your written request. We will keep using the PHI already collected about you. It will only be used to complete the research and maintain the scientific integrity of the study.

APPROVED
MARCH 09, 2018
Methodist Healthcare System
Institutional Review Board

SIGNATURE PAGE FOR INFORMED CONSENT

I confirm that the study has been explained to me in a language that I am able to fully understand.

My signature below signature shows that:

- I volunteer to take part in this study.
- I have read, or have had read to me, the information in this form and believe I understand the procedures, risks and benefits of this study.
- I authorize the collection, use and disclosure of my PHI as described in this form.
- I have had a chance to ask questions, and all of my questions have been answered to my satisfaction.
- I do not give up any of my rights by taking part in this study.
- I understand that I will not receive any payment in any way for my part in this study.
- I understand that my information from the study will be held in both computerized and manual filing systems. These files will not identify me by name. These records may be used for product registration. They may be made available to health authorities worldwide. They may be sent out of the United States for processing.
- I will receive a copy of this signed form.

I understand that I am free to withdraw from the study:

- at any time
- without having to give a reason for withdrawing, and
- without affecting my future medical care.

Patient Signature ___________________________________ Date ______________

Patient Name (please print) ____

<u>Name and signature of Person Conducting Informed Consent discussion</u>

I, the named and undersigned, have fully explained the relevant details of this study to the patient named above.

Consent Discussion _____________________________________ Date _______________

Patient Name (please print) ____

RSHCN

APPROVED
MARCH 09, 2018
Methodist Healthcare System
Institutional Review Board
